# Filtration and Normalization of Sequencing Read Data in Whole-Metagenome Shotgun Samples

**DOI:** 10.1371/journal.pone.0165015

**Published:** 2016-10-19

**Authors:** Philippe Chouvarine, Lutz Wiehlmann, Patricia Moran Losada, David S. DeLuca, Burkhard Tümmler

**Affiliations:** 1 Department of Pediatrics, Baylor College of Medicine, Houston, Texas 77030, United States of America; 2 Clinical Research Group, ‘Molecular Pathology of Cystic Fibrosis and Pseudomonas Genomics’, OE 6710, Hannover Medical School, Hannover D-30625, Germany; 3 Biomedical Research in Endstage and Obstructive Lung Disease (BREATH), German Center for Lung Research, Hannover, Germany; University of Westminster, UNITED KINGDOM

## Abstract

Ever-increasing affordability of next-generation sequencing makes whole-metagenome sequencing an attractive alternative to traditional 16S rDNA, RFLP, or culturing approaches for the analysis of microbiome samples. The advantage of whole-metagenome sequencing is that it allows direct inference of the metabolic capacity and physiological features of the studied metagenome without reliance on the knowledge of genotypes and phenotypes of the members of the bacterial community. It also makes it possible to overcome problems of 16S rDNA sequencing, such as unknown copy number of the 16S gene and lack of sufficient sequence similarity of the “universal” 16S primers to some of the target 16S genes. On the other hand, next-generation sequencing suffers from biases resulting in non-uniform coverage of the sequenced genomes. To overcome this difficulty, we present a model of GC-bias in sequencing metagenomic samples as well as filtration and normalization techniques necessary for accurate quantification of microbial organisms. While there has been substantial research in normalization and filtration of read-count data in such techniques as RNA-seq or Chip-seq, to our knowledge, this has not been the case for the field of whole-metagenome shotgun sequencing. The presented methods assume that complete genome references are available for most microorganisms of interest present in metagenomic samples. This is often a valid assumption in such fields as medical diagnostics of patient microbiota. Testing the model on two validation datasets showed four-fold reduction in root-mean-square error compared to non-normalized data in both cases. The presented methods can be applied to any pipeline for whole metagenome sequencing analysis relying on complete microbial genome references. We demonstrate that such pre-processing reduces the number of false positive hits and increases accuracy of abundance estimates.

## Introduction

Metagenomics is the study of microbial communities in their natural habitat without isolation or cultivation of individual species [1]. The boom of next-generation sequencing technologies makes it more affordable to sequence whole metagenomes of environmental samples with high coverage. This technique is known as whole-metagenome shotgun (WMS) sequencing. Declining sequencing costs should make it an attractive alternative to traditional 16S rDNA, RFLP, or culturing approaches. Techniques based on biomarkers rather than whole-genome analysis can suffer from inaccuracies due to copy number variation or lack of sufficient sequence similarity between the primers and their targets. Moreover, WMS sequencing allows direct inference of the metabolic capacity and physiological features of the studied metagenome [2,3]. In addition, the WMS sequencing approach allows estimation of fungi and viruses in the sample, which is not possible with the biomarker-based metagenomic techniques. The sequencing coverage of individual bacterial genomes comprising the metagenome will vary based on two factors: their abundance in the sample and sequencing factors. Sequencing factors include GC bias, fragmentation bias, sequencing depth, sequencing protocols, etc. Normalization of these biases can be used for correct estimation of bacterial abundance in the sample. Another pitfall in reporting bacterial abundance using the WMS approach is related to local spikes in sequencing coverage. Reads that are clustered only in a few loci of a single bacterial hit as opposed to being randomly distributed are most likely located in genomic islands originating via horizontal transfer. The true origin of these reads is the source of the genomic island rather than the organism containing this genomic island; therefore, such bacterial hits must be filtered out prior to identification and quantification.

Finally, lengths of the bacterial reference genomes also contribute to the likelihood of these genomes being sequenced. The need for such length correction has been previously addressed in RNA-seq applications, in which gene lengths affect the number of cDNA reads representing the expression levels of these genes [4]. For completeness, it should be mentioned if a bacterial cell is dividing faster than the time necessary for chromosome replication, it inherits a chromosome that contains replication forks. At maximal growth rates, the measured amount of DNA per cell is three to four genome equivalents.[5]. However, accounting for this phenomenon is currently an unresolved issue; therefore, it was not addressed in our normalization procedure.

The WMS sequencing approach has been used since short read NGS platforms became available. For example, Breitbart *et al*. [6] used this approach to assess viral diversity in ocean water in 2002. Since then a plethora of tools have been developed for analysis of WMS data [7–16]. A short overview of most commonly used whole metagenome microbiome profiling tools is provided in S1 Table. However, the methods implemented in these tools failed to address the GC bias problem and only a few of them address the issues of length difference of the reference sequences [7,13,16]. To our knowledge only the Genometa pipeline [9] currently implements a check for potential false positive hits due to clustered genomic island reads, without addressing the first two problems (GC bias and length difference of the references).

In this paper we present filtration and normalization procedures meant to improve accuracy in estimation of bacterial abundances in WMS samples. This implementation relies on the assumption that complete genomic sequences are available as references for most microorganisms of interest, which is often the case, in medical diagnostics. For species level analysis, the aim is to classify each read by its species of origin or alternatively to discard it if its origin is ambiguous or misleading. Each classified read is given a weight based on its GC content and the GC content of its hit (the bacterial genome to which it is assigned). Finally, the length of the hit is used to normalize the GC-weighted counts for accurate abundance estimation.

In our approach, we utilize reference genome mapping to attribute reads to specific species. Although relying on reference genomes does prohibit the discovery of unknown or poorly characterized species, the majority of clinically relevant bacteria are well characterized. In comparison to culture based methods used in the clinic, the current reference genome data allow for more comprehensive characterization. Furthermore, the GC bias correction as a methodology is largely independent of the mapping strategy and is applicable to reference-free approaches.

In the Filtration Methods section, we present two complementary methods for filtration of clustered reads potentially mapped to genomic islands. The first method is based on a single-sample t-test of the mean distances between the reads mapped to the same reference genome. The second method uses the uniform distribution model to estimate the genome size of the reference based on the distances among all reads mapped to it. The difference between the actual and estimated genome sizes allows us to conclude whether the read mapping locations are spread enough in order not to come from genomic islands incorporated into this reference genome.

In the GC Normalization section, we present a GC bias model that was created using non-linear regression from the empirical data collected by sequencing samples of seven bacteria with various GC contents using the SOLiD 5500xl technology. Similar models should be created for each sequencing platform as the GC biases are expected to vary for each of them [17].

In the following sections we discuss various topics related to estimation of bacterial abundances. In particular, we concentrate on dealing with reads mapping equally well to multiple locations in the same or multiple genomes (for the species-level analysis), genome length normalization, and bacterial load assessment.

Finally, we tested our normalization procedures on two validation sets containing pools of eight bacteria mixed in different predetermined concentrations and sequenced from 250 ng and 20 ng of bacterial DNA respectively. Most of the bacteria in the validation pools were not used in the samples for generation of the GC normalization model. After application of our normalization procedures we observed four-fold reduction in root-mean-square error (RMSE) in both cases.

## Results and Discussion

### Filtration of hits with clustered reads

Bacterial genomes are known to actively recombine and incorporate genomic islands from bacteria of other strains or species. This can confound correct identification of species in a metagenomic sample. To prevent reporting false positive bacterial hits that are identified exclusively by reads mapping to genomic islands, it is necessary to filter out such bacterial hits that only have a few clusters of mapped reads. To achieve this, we use a single-sample t-test of the read start differences of neighboring reads. Ideally, all reads mapped to a genome should be uniformly distributed across its length. While some sequencing biases, such as GC bias or DNA fragmentation bias during the library preparation can distort a perfect uniform distribution of the read positions, such distortions are still negligible compared to the location bias of the reads mapping exclusively to the genomic islands. We can formally test it by calculating distances between the read start positions Δ of the neighboring reads and performing a single-sample t-test of the difference of the actual mean of such distances $\bar{\Delta }$ and the null hypothesis mean ${\bar{\Delta }}_{H0}=G/N$, where *G* is the genome length and *N* is the number of mapped reads. Finding the correct p-value threshold for the test can be assisted by printing out a table of counts of distances between the starts of the neighboring reads for each filtered and unfiltered bacterial genome. This method can also be augmented by heuristics that filter out bacterial hits based on setting an upper limit on the percent of allowed short read distances, e.g., less than 10 bp, or based on estimation of a bacterial genome size, as specified by Davenport *et*.*al* [9] (supplementary materials) and comparing it with the actual genome size (S1 File).

We tested this approach to filtering hits with reads potentially clustering in genomic islands by collecting percentages of filtered out reads in 30 metagenomic samples (sputum of cystic fibrosis patients). If our method was filtering hits (and the associated reads) in samples with fewer reads more aggressively, this would indicate that our approach is biased by the sample read count and is not applicable. However, we only found weak correlation between the number of mapped reads and the proportion of filtered out reads (Pearson’s *R* = -0.237). The distribution of percentages of the filtered out reads vs. the number of mapped reads is shown in S1 Fig. The ability to detect islands is dependent on the island coverage. Using simulations, we observe that as little as three reads in the genomic island area are sufficient for detection, with a p-value of less than 10^−3^ (S2 Fig).

### GC normalization

**Fig 1:**
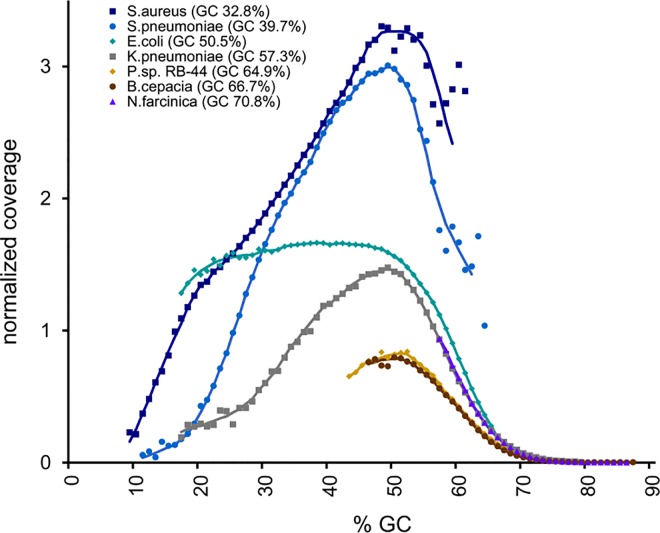
Normalized coverage vs. GC content of seven bacteria. The sequenced bacteria are: *Staphylococcus aureus* (dark blue line, 32.8% GC), *Streptococcus pneumoniae* (light blue line, 39.7% GC), *Escherichia coli* (teal line, 50.5% GC), *Klebsiella pneumoniae* (gray line, 57.3% GC), *Pandoraea sp*. (tan line, 64.9% GC), *Burkholderia cepacia* (brown line, 66.7% GC), and *Nocardia farcinica* (purple line, 70.8% GC). Equal amounts of DNA from each bacterium were sequenced and mapped to their respective genome references. The normalized coverage was calculated for each GC percentage point based on the proportion of the number of reads mapped to 100 bp genome windows having this GC content to the number of such windows. These values were normalized by the proportion of the number of all mapped reads to the number of all windows for a given genome.

GC bias is the most significant bias adversely affecting coverage of GC-rich regions of a sequenced genome. Generally, G and C bonds are more stable than A and T, due to the fact that they have one extra hydrogen bond and their stacking interactions are quite different. Particularly, G-C pairing does not affect DNA duplex, while A-C pairing is always destabilizing [18]. It is present to various degrees in short-read next-generation sequencing technologies [17]. Obviously, GC bias affects abundance estimates of bacterial genomes in metagenomic samples, particularly the ones with high GC content. As noted in [19] GC of the full PCR-amplified fragment rather than the forward read (or forward and reverse reads for paired-end sequencing) of this fragment primarily determines the GC bias. This also confirms previous findings of Aird *et al*. [20] that the PCR component of the GC bias is the one that contributes most, while the downstream instrument related bias is also present, but to a lesser degree. Moreover, there exists a global source of GC bias on the scale larger than the fragment length due to association with higher order structures of the DNA [19]. We take into account this global source of GC bias and the PCR-induced, fragment GC bias by considering GC content of the genome to which the read is mapped, which is important in metagenomic samples with multiple bacterial genomes of a wide GC range. We take into account the post-PCR instrument GC bias by considering GC content of each read. In other words, a GC-rich read from a GC-poor locus typical of GC-poor genomes is more likely to be sequenced than a read with the same GC content, but located in a GC-rich locus. We confirmed this idea by sequencing equal amounts of seven bacteria ranging in GC content from 32.8 to 70.8% and calculating a GC bias curve for each of them (Fig 1). The curves in Fig 1 were created by calculating the normalized coverage (GC bias) at each GC percentage point *i* using the CollectGcBiasMetrics utility from Picard Tools (http://broadinstitute.github.io/picard/). This program calculates normalized coverage for the case of sequencing a single genome as follows:

$$NormCov_{i}=\frac{Rst_{i}}{W_{i}}/\frac{Rst_{Total}}{W_{Total}},$$ (1)

where *Rst*_*i*_ is the count of read starts within windows of GC% *i* and *W*_*i*_ is the count of windows of GC% *i*. The ratio with the total values (*Rst*_*Total*_/*W*_*Total*_) normalizes the estimation to the average number of reads per window across the whole genome. To calculate normalized coverage of a single bacterium in a pooled sample we modified this formula as follows:

$$NormCov_{ij}=\frac{Rst_{ij}}{W_{ij}}/\frac{Rst_{Total}/m}{W_{Totalj}},$$ (2)

where *j* = 1,…, *m* are indices for *m* bacteria in a pooled sample. In this formula we distribute the total number of read starts in a sample *Rst*_*Total*_ equally among all bacteria in the pool instead of using the actual number of mapped reads to bacterium *j*, thus, accounting for potential under- or over representation of bacteria due to their GC content. However, the total number of windows of various GC content is unique to a particular genome, therefore, *W*_*Totalj*_ is used in the formula. The read length of our sequences was 75 bp, though some reads were trimmed during the alignment. We used the default window size for short reads, which is 100 bp.

As shown in Fig 1, reads from GC-poor genomes were overrepresented, while the general bell-shaped-like curve of GC bias can be, at least partially, observed in all genomes. To develop our GC normalization model we created several linear regression models of various complexity as well as a non-linear regression model that incorporates a Gaussian exponential function to better approximate the expected bell shapes of the observed normalized coverage curves. When applied to our validation data this non-linear multiple regression model produced bacterial abundance estimates with significantly lower root-mean-square error (RMSE) compared to simpler linear models (Table 1), therefore, it was chosen for our normalization procedure. In this model the output variable is the normalized coverage coefficient of the read (as defined by the equation above), while the GC content of the read and the GC content of the genome to which this read mapped are the input variables. To normalize for GC bias each read should be divided by its normalized coverage coefficient.

**Table 1:** Regression models considered for GC normalization. We explored several regression models, from simple linear models using only one input variable (genome GC content) to more complex by progressively increasing the number of terms and using two input variables (read GC content and genome GC content). While this strategy helped us find models with lower RMSE, it eventually led to overfitting and a significant increase in RMSE (the forth-degree polynomial model). However, using non-linear regression with a Gaussian exponential term significantly improved RMSE (last model). Complete results of model testing with estimates of abundance of each bacterium in the validation sets are provided in S2 Table. R output with statistics for the tested models is included in S2 File.

Model of normalized coverage	Unique solution found	Residual standard error	Degrees of freedom	RMSE (Experiment 1, 250 ng bacterial DNA), %	RMSE (Experiment 2, 20 ng bacterial DNA), %
*θ*_1_ + *θ*_2_ log(*GC*_*G*_)	Yes	0.6519	340	8.35	8.51
*θ*_1_ + *θ*_2_*GC*_*G*_ + *θ*_3_*GC*_*G*_^2^	Yes	0.6515	339	8.09	8.26
*θ*_1_ + *θ*_2_*GC*_*R*_ + *θ*_3_*GC*_*R*_^2^ + *θ*_4_ log(*GC*_*G*_)	Yes	0.4414	338	8.41	8.69
*θ*_1_ + *θ*_2_*GC*_*R*_ + *θ*_3_*GC*_*R*_^2^ + *θ*_4_*GC*_*R*_^3^ + *θ*_5_ log(*GC*_*G*_)	Yes	0.373	337	6.24	6.53
*θ*_1_ + *θ*_2_*GC*_*R*_ + *θ*_3_*GC*_*R*_^2^ + *θ*_4_*GC*_*R*_^3^ + *θ*_5_*GC*_*R*_^4^ + *θ*_6_ log(*GC*_*G*_)	Yes	0.3441	336	11.7	11.92
$\theta_{1}e^{-0.5{\left(\frac{GC_{R}-\theta_{2}}{\theta_{3}}\right)}^{2}}+\theta_{4}+\theta_{5}\log(CG_{G})$	No	NA	NA	NA	NA
$\theta_{1}e^{-0.5{\left(\frac{GC_{R}-\theta_{2}}{\theta_{3}}\right)}^{2}}+\theta_{4}+\theta_{5}GC_{R}+\theta_{6}GC_{R}{}^{2}$	No	NA	NA	NA	NA
$\theta_{1}e^{-0.5{\left(\frac{GC_{R}-\theta_{2}}{\theta_{3}}\right)}^{2}}+\theta_{4}+\theta_{5}GC_{R}+\theta_{6}GC_{R}{}^{2}+\theta_{7}\log(GC_{G})$	No	NA	NA	NA	NA
$\theta_{1}e^{-0.5{\left(\frac{GC_{R}-\theta_{2}}{\theta_{3}}\right)}^{2}}+\theta_{4}+\theta_{5}GC_{R}+\theta_{6}GC_{R}{}^{2}+\theta_{7}GC_{R}{}^{3}+\theta_{8}\log(GC_{G})$	Yes	0.3283	334	2.87	2.91

The following formula was used for the regression:

$$NormCov(GC_{R},GC_{G};\theta )=B(GC_{R};\theta_{1},\theta_{2},\theta_{3},\theta_{4})+\theta_{5}GC_{R}+\theta_{6}GC_{R}{}^{2}+\theta_{7}GC_{R}{}^{3}+\theta_{8}\log(GC_{G}),$$ (3)

where *GC*_*R*_ is GC content of the read, *GC*_*G*_ is GC content of the genome to which the read is mapped, *θ*_1,…,8_ are regression coefficients, and *B* is the Gaussian exponential function of read GC content, defined as

$$B(GC_{R};\theta_{1},\theta_{2},\theta_{3},\theta_{4})=\theta_{1}e^{-0.5{\left(\frac{GC_{R}-\theta_{2}}{\theta_{3}}\right)}^{2}}+\theta_{4}.$$ (4)

**Fig 2:**
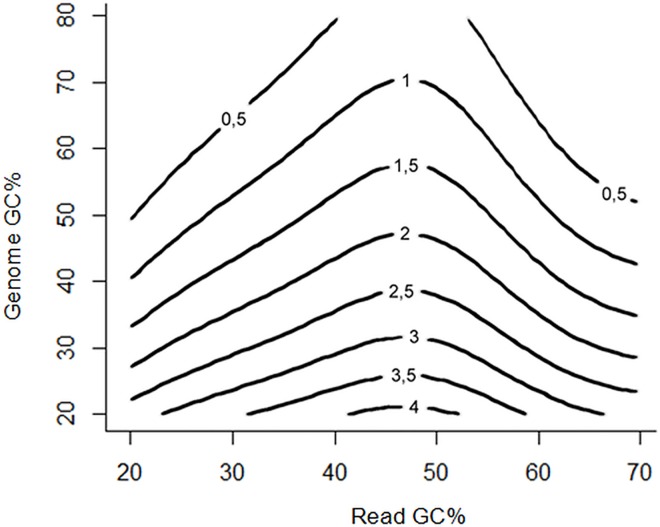
Contour plot of the proposed GC normalization model. The model approximates expected normalized coverage of genomic regions stratified by their GC content for bacterial genomes of various overall GC content in a pooled whole-metagenome shotgun sequencing sample. Generally, the regions higher than the contour line of value 1 are overrepresented and the regions lower than this line are underrepresented. To perform the GC normalization, each binned read in a metagenomic sample should be divided by the normalized coverage value predicted based on its GC content and the content of the genome to which it mapped.

The third degree polynomial in the regression formula accounts for imperfections in the bell shapes of the observed normalized coverage curves. The last term of the formula approximates the observed influence of genome GC content. The observed abundance drops as the corresponding genome GC content increases. However, this relationship is less evident in the genomes with higher GC contents. For example, there is no significant difference between the curves of *Pandoraea sp*., GC 64.9% and *Burkholderia cepacia*, GC 67.7%, and in the case of the *Nocardia farcinica* genome with the highest GC content (70.8%) there is even a slight increase in normalized coverage values. Therefore, we used the (upside-down) logarithm function to approximate this dependency. The constructed model is only an approximation, therefore, to avoid extremely low predicted values, which would set unreasonably high weights to some reads, we set all predicted values less than 0.01 to 0.01. The contour plot of the produced model is shown in Fig 2.

The effects of GC- and genome length normalization are shown in S3 Table where the species abundances of a sample are reported as raw read counts, GC-weighted read counts, and GC-weighted read counts per Mb of reference. The three reported counts vary significantly for some species changing their rank number after the normalization steps.

### Reference genome length normalization

Another important consideration for accurate reporting of bacterial abundances in metagenomic species is the genome length normalization. This procedure is common in other analyses involving counting of short reads mapped to genomic features, e.g., RNA-seq, where RPKM [4] values are used for absolute levels of gene expression. Following the same logic, longer bacterial genomes will have a higher chance to produce sequencing reads. To account for this, the final bacterial abundances can be reported as GC-weighted read counts per Mb of reference. The last column in S3 Table shows such values.

### Multireads

From our experience, depending on the promiscuity of bacteria present in a metagenomic sample the percentage of multireads, i.e., the reads that map to multiple locations in the same or multiple genomes, can vary from 5 to 96% of the total number of reads. Therefore, to perform the species-level analysis, the multireads can be used for abundance estimates only if each of them is counted only once and assigned to a single species. Our in-house Perl script (S3 File) performs such assignment by discarding multireads that map to more than one species and collapsing hits to multiple strains of the same species produced by a single read.

### Validation of the GC and genome length normalization model

To test performance of our GC and genome length normalization procedures we created two validation sets containing pools of eight bacteria most of which were not used in the samples for generation of the GC normalization model. In each pool the bacteria were mixed in different concentrations in human DNA background. The DNA amounts for each bacterium in these pools are shown in S2 Table. This table also contains the counts of raw reads mapped to each genome reference for bacteria present in the sample and GC normalized read counts per Mb or reference generated by various regression models that we tested. To measure the overall performance of these models for each of the experiments we used root-mean-square error (RMSE), which is calculated as the square root of the mean value of the squared residuals. The best performing model utilized multiple non-linear regression and afforded RMSE reduction from 11.63 to 2.87% (Experiment 1, the pool that was sequenced from 250 ng of bacterial DNA) and from 11.9 to 2.91% (Experiment 2, the pool that was sequenced from 20 ng of bacterial DNA) compared to estimates based on raw read counts. In both cases the observed RMSE reduction was four-fold. This shows that the normalization effects are reproducible and tolerate the difference in amounts of sequenced DNA in the tested range.

**Fig 3:**
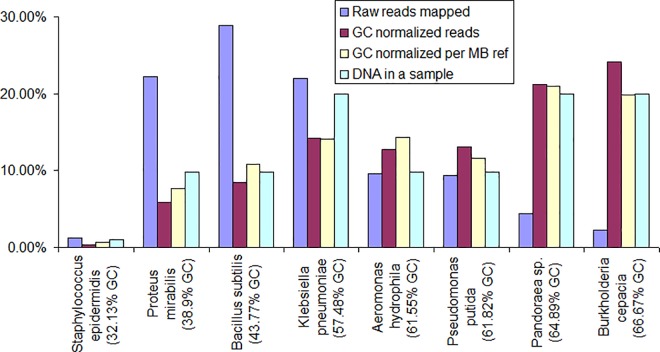
Estimated relative abundances (Experiment 1). Abundance of each bacterium in the experiment is represented by four bars. The first (left) bar is abundance estimated by raw reads mapped to the genomic reference. The second bar is abundance corrected for GC bias. The third bar is the GC-bias-corrected abundance per Mb of reference. The forth bar is the actual abundance based on the amount of DNA in the pooled sample. In most cases the corrected values follow the actual values much closer than those based on raw reads.

A graphical representation of GC and length bias correction is illustrated in Fig 3, which is based on the data from Experiment 1. There the bacteria are ordered by their genome GC content and the histogram shows percentages of raw reads, normalized reads, and DNA amounts. It can be seen that the abundance estimates based on raw reads alone would overestimate the bacteria with lower mid-range GC content and significantly underestimate the bacteria with in the higher GC content range. In most cases the estimates based on the normalized read counts follow the actual DNA distribution much closer.

### Bacterial load estimates

**Fig 4:**
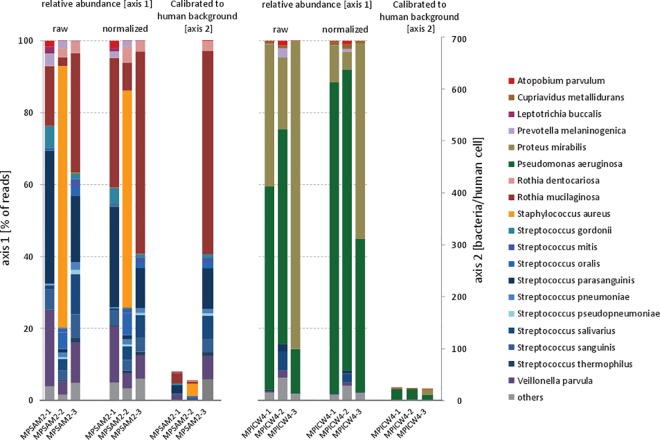
Relative bacterial abundances and bacterial abundances per human cell in upper airways of two cystic fibrosis patients measured at three time points. The two bar charts represent bacterial abundance estimates in sputa sampled from two subjects with cystic fibrosis (CF) who are homozygous for the *p*.*Phe508del* mutation in the *CFTR* gene. Each chart contains three sets of three bars, where the bars in each set represent three samples which were taken in 3-month intervals. The first two sets represent relative abundances based on raw read counts (first set) or based on GC and genome length normalized counts (second set). The third set of bars shows bacterial abundances per human cell based on the normalized counts in the second set calibrated to human background. The patient on the left (MPSAM2) has significantly increased absolute bacterial abundance (per human cell) in the third sample, which is not obvious from the relative abundance histograms.

Finally, estimation of bacterial loads in a metagenomic sample may be desirable, e.g., to assess an infection in a body site. Within the context of infectious disease or clinical microbiology, bacterial load is defined as the total amount of CFU/mL clinical specimen (e.g., ml sputum). In order to express bacterial load in terms of sequencing reads we can define it as the total of absolute bacterial abundances of all detected bacteria per host cell. In this case, we can utilize the host background DNA to do this assessment and report absolute bacterial abundances per DNA content of a single host cell. If the host is human, the absolute abundance of each species per human cell can be calculated as follows:

$$A=\frac{6191.39C_{GCperMbR}}{C_{H}},$$ (5)

where *C*_*GCperMbR*_ is the GC weighted read count per Mb reference of the given species, *C*_*H*_ is the human read count, and 6191.39 is the length of a diploid human genome divided by a million (to account for the bacterial count scale). Of note, this only provides a general estimation that can be confounded by various factors during library preparation or presence of free DNA in the sample. However, such estimates would still be informative in reproducible conditions, e.g., for tracking disease progression over time. To illustrate this point, Fig 4 shows relative and absolute (per human cell) abundances of bacteria in sputa taken from two patients with cystic fibrosis at 3-month intervals. Please note that the presentation of relative percentages as it is common for most microbiome studies may lead to an erroneous interpretation.

## Conclusions

Estimation of bacterial abundances by whole-metagenome shotgun (WMS) sequencing is based on the counts of reads mapped to a collection of entire bacterial genome references rather than a region of the 16S gene or other biomarkers uniquely identifying a genome. This poses a challenge since some reads mapped to one of the references can belong exclusively to genomic islands horizontally transferred from other organisms. Other challenges include uneven coverage of the reference genomes due to GC bias inherent to PCR and short-read next-generation sequencing platforms. Even varying lengths of the reference genomes affect likelihood of a read mapping to a particular bacterial genome reference, which is not a problem for 16S analysis where the sequenced variable regions of the 16S gene are all of the same length. However, we have shown that all these obstacles can be addressed by filtration and normalization procedures, thus leading to more accurate estimation of bacterial abundances in a metagenomic sample.

The structure of bacterial genomes is often dependent on frequent recombination due to significant evolutionary pressure to survive in hostile environments [21,22]. Some bacterial genomes are particularly promiscuous in accepting horizontally transferred genomic islands, e.g., *Pseudomonas* [23,24] or *Burkholderia* [25]. We have shown that it is possible to identify false positive bacterial hits with reads clustered in their genomic islands. Application of our filtration procedure removed 1.7 to 15.9% of such bacterial hits in the 30 cystic fibrosis airway samples used for this study.

Typical methods for GC correction rely on applying local regression [19,26–28], e.g., LOESS, or quantile normalization methods [28,29] to the data points created by read counts mapped to genes or non-overlapping windows of the reference sequence grouped into GC-stratified bins vs. the GC content of the bins. The raw read counts are then normalized, e.g., by calculating the correction value for each feature as the difference between the fitted value and the median across all bins. This strategy works well when a single genome is analyzed. In a metagenomic sample certain genomes have a small number of reads mapped to them making this approach inapplicable for GC normalization of actual data. On the other hand, it is possible to create an approximation model based on a collection of GC bias curves of genomes varying in their GC content (Fig 1). We do not rely on LOESS regression, because it requires large and densely sampled datasets covering the entire two-dimensional parameter space (read GC and genome GC). In our case due to technological limitations we did not have data for very low GC regions of the high-GC genomes or very high GC regions of the low-GC genomes (Fig 1). Therefore, we used multiple nonlinear regression that specified the expected bell-shape curve of the regions with the missing data. Moreover, the resulting regression function can be easily implemented in a script and applied to metagenomic data generated using the same DNA sequencing setup. It is important to note that the overall GC bias is unique to a particular sequencing setup and comes primarily from the PCR GC bias resulting from the kits used and the downstream instrument GC bias, which is different for different platforms. For example, Illumina HiSeq and 5500xl SOLiD platforms have significantly different GC bias as shown in [17]. Therefore, metagenomic labs interested in implementation of our GC bias normalization procedure should collect the data for individual bacterial genomes of varying GC content and repeat our regression procedure to identify the regression coefficients specific to their sequencing setup.

Finally, GC normalized reads can be reported per Mb of bacterial reference to account for the increased likelihood of mapping reads to longer genomes. Batch effects can also lead to bias associated with the length of the reference sequence, e.g., in RNA-seq samples. However, it has been reported that that this type of length effect is the strongest in features less than 1000 bp and it plateaus after 5000 bp [29], therefore, it does not affect estimation of bacterial abundances in metagenomic data.

In some cases, metagenomic samples of human microbiota taken over a certain period of time from the same source, e.g., sputum of a cystic fibrosis patient, afford an opportunity to report changes in bacterial load. In this case, the proportion of human DNA background can be utilized to calculate the total bacterial load, which can reveal the disease stage of the patient. The relative bacterial abundances estimated as described above can be transformed to absolute estimates based on the identified bacterial load.

## Methods

### DNA library preparation

Seven bacterial reference strains with different GC contents were obtained from the American Tissue Culture Collection or the in-house collection: *Burkholderia cepacia* (67% GC, ATCC 25416), *Escherichia coli* (50% GC, ATCC 25922), *Klebsiella pneumoniae* (57% GC, ATCC 10031), *Nocardia farcinia* (71% GC, MHH 442780), *Pandoraea sp*. (64.9% GC, RB-44), *Staphylococcus aureus* (33% GC, ATCC 25923), *Streptococcus pneumonia* (40% GC, ATCC 49619). Bacteria were grown until exponential phase in LB broth. DNA was isolated from the bacteria with the DNeasy kit (QIAGEN) following the instructions of the manufacturer. Yield of double-stranded DNA was quantified with the Qubit spectrofluorimeter (Invitrogen). Aliquots of five ng of each bacterial DNA preparation were added to 315 ng human DNA in a total volume of 130 μl low TE-buffer (Life Technologies).

Induced sputum was collected from subjects with cystic fibrosis during inhalation with aqueous hypertonic saline (6% v/v NaCl). The sputum sample was diluted 1:4 with phosphate-buffered saline/2% (v/v) mercaptoethanol at 4°C and incubated under shaking for 2 h on ice. The specimen was centrifuged at 3,800 g for 15 min at 10°C. After removal of the supernatant, the pellet was dried and then dissolved in 10 ml distilled water for 15 min at 4°C. The suspension was again centrifuged (3,800 g, 15 min, 10°C), the precipitate was dissolved in distilled water for 15 min at 4°C, pelleted and the pellet was transferred into an Eppendorf tube for incubation with DNase I (0.42 mL H2O + 50 μL RD buffer (QIAGEN) + 35 μL DNase I) at 30°C for 90 min under shaking. The suspension was added to 10 ml SE-buffer and washed three times with 10 mL SE each by precipitation (3,800 g, 15 min, 10°C). The pellet was dissolved in 0.5 mL SE in an Eppendorf tube and precipitated again (12,000 g, 10 min, 10°C). DNA was extracted from this pellet with the Nucleo Spin Tissue Kit (Macherey & Nagel) by following the hard-to-lyse-bacteria protocol and stored at 4°C in TE buffer at 4°C until use. Sample collection was approved by the Ethics Committee of Hannover Medical School, Germany (Study no. Ha110/07; date of approval 19-July-2012). Participants provided written informed consent. In case of minors parents and the study participant both signed the 3-page patient information. The documents of written participant consent were stored in the office of the responsible study physician. The study physician performed the pseudonymization of study materials and clinical data.

Preparation of fragment libraries and sequencing were performed at the E120 scale according to the protocols provided by Thermo Life Technologies for SOLiD5500 instruments (generation of libraries: https://tools.lifetechnologies.com/content/sfs/manuals/4460960_5500_FragLibraryPrep_UG.pdf; emulsion PCR: Emulsifier, Amplifier http://tools.lifetechnologies.com/content/sfs/manuals/cms_102275.pdf; Enricher http://tools.lifetechnologies.com/content/sfs/manuals/cms_089261.pdf). All sequencing data generated for this project were deposited to NCBI SRA database with accession number SRP075586.

### Analysis workflow

Filtration and normalization methods presented in this paper can be applied to any WMS analysis pipeline that relies on mapping individual reads to complete genome references. For completeness, here we present our analysis pipeline.

The SOLiD reads of metagenomic samples are first trimmed to variable lengths (no shorter than 45 bp) to have at least 40 bases with Q> = 20. The trimmed reads are checked for contamination by Homo sapiens DNA by aligning them against the “1000 Genomes” Homo sapiens reference, which includes contigs unassigned to chromosomes, using the ultrafast Bowtie2 aligner [30] to speed up the processing. The unaligned reads are corrected using SOLiD’s SAET utility, which increases the number of mapped reads by 40–50% in genomes of size 1Kbp - 200Mbp with coverage 10-4000x and read length 25–75bp (according to the manufacturer), however, the correction does not lead to significant improvements in mapping to large genomes, e.g., human genome. The corrected reads are aligned against available reference genomes of bacteria, viruses, fungi, and known contaminates using the Novoalign (http://www.novocraft.com/) short read aligner. We set the–r parameter of Novoalign either to All (for the species level analysis) or to None (for the strain level analysis). This parameter determines the multiread strategy as described in the Other Considerations section below. Reads aligned to the bacterial references are filtered out if they do not pass the clustering tests. The reads are normalized to correct the GC bias and reported as weighted counts per Mb of reference. The unaligned reads can still belong to species without reference genomes. These reads can be used for functional analysis after contig assembly with the subsequent blastx Uniprot search.

### GC normalization

To identify the effect of the GC bias on abundance estimates of a collection of genomes in a metagenomic sample we sequenced samples of seven bacterial genomes. Fig 1 shows superimposed curves of normalized coverage vs. GC percentage for each of the genomes. All data points with p-values less than 5% were removed.

The normalized coverage reflects the GC bias of the genome at locations stratified by each GC percentage point. While the curves vary in shape, they all follow the same unimodal bell-curve pattern with various degrees of distortion. Notably, the *E*. *coli* curve (genome GC 50.5%) remains relatively high in the GC range from 25 to 50%. This is determined by the shape of the expected GC curve, which can be constructed by counting the number of windows with a particular GC content in the genome. This variation in GC curve shapes is captured in the regression model that we present. Another clear observation from Fig 1 is that there is an inverse relationship between normalized coverage and genome GC content. Therefore, our GC bias model was designed with two input variables: read GC content and GC content of the genome to which the read was assigned. The model was created using multiple non-linear regression implemented in the nls function in R as described in Results and Discussion. For our normalized coverage data, presented in Fig 1, the residual standard error was 0.3283 on 334 degrees of freedom. Conversion was achieved after 12 iterations with the conversion tolerance of 9.335e-06. The contour plot of the model is shown in Fig 2. All values of the output variable approximated below 0.01 are programmatically set to 0.01 to avoid extremely high weights assigned to a single read. The read weights are calculated as reciprocals of normalized coverage determined by the model. Less complex linear regression models that were not selected due to higher RMSE (Table 1) were created using the lm function in R.

## Supporting Information

S1 Fig Percentage of filtered out reads vs. the total number of mapped reads. Read alignments of thirty cystic fibrosis samples were filtered to remove hits with reads mapped to horizontally transferred genomic islands. There is no significant correlation between the sample size and the percentage of filtered out reads.
(TIF)

S2 Fig Effect of coverage of genomic islands on detection. Genomic islands were simulated assuming a uniform distribution of reads at various levels of coverage. The simulation parameters assumed a genome length of three million bases, island size of 1000 bases and no coverage in non-island regions. The negative log of the p-value used for detecting genomic islands increases with coverage. As little as 3 reads are necessary for detection with significant p-values (p < 2.7x10^-4^).
(PNG)

S1 File Sample output file. The first section of the output contains a table with bacterial abundance estimates including raw read counts, GC-corrected read counts, and GC-corrected read counts per Mb of reference. The subsequent sections contain uncertain bacterial hits where the upper bound of the genome size estimate is 10 to 50% of the actual size. The t-test statistics and especially the counts of distances between read starts of the neighboring reads can be helpful in manual determination of whether or not the reads aligned to this hit are clustered in genomic islands. In these cases, prior domain knowledge about the sample can tip the decision one way or another.
(RTF)

S2 File R output containing statistics of GC-normalization models (RTF)

S3 File Perl script for filtration of genomic islands and normalization by GC content and genome length (ZIP)

S1 Table Comparison of commonly used whole-metagenome microbiome profiling methods with the proposed method (DOC)

S2 Table Top 30 bacteria found in sputum of a cystic fibrosis patient (XLS)

S3 Table Raw and normalized read counts for bacteria in two validation experiments (DOC)
