# Supplementary figures and images for: Filtration and Normalization of Sequencing Read Data in Whole-Metagenome Shotgun Samples
Source: PLoS One. 2016 Oct 19;11(10):e0165015. doi: 10.1371/journal.pone.0165015 (PMC5070866; doi:10.1371/journal.pone.0165015)

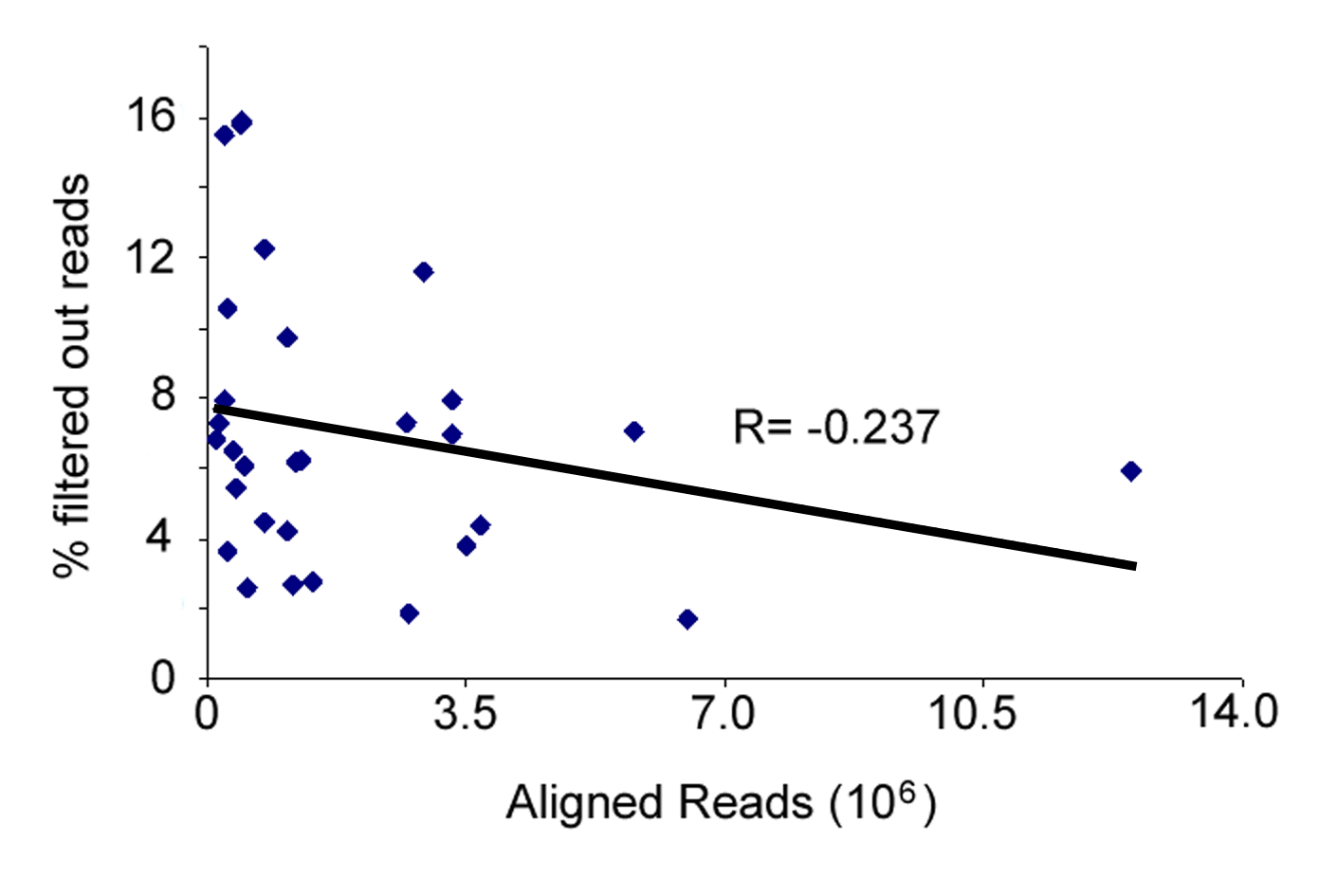

Supplement: S1 Fig — Read alignments of thirty cystic fibrosis samples were filtered to remove hits with reads mapped to horizontally transferred genomic islands. There is no significant correlation between the sample size and the percentage of filtered out reads. (TIF) [file pone.0165015.s001.tif]

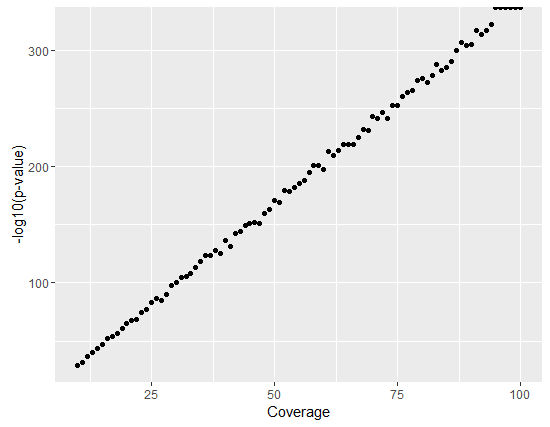

Supplement: S2 Fig — Genomic islands were simulated assuming a uniform distribution of reads at various levels of coverage. The simulation parameters assumed a genome length of three million bases, island size of 1000 bases and no coverage in non-island regions. The negative log of the p-value used for detecting genomic islands increases with coverage. As little as 3 reads are necessary for detection with significant p-values (p < 2.7x10^-4^). (PNG) [file pone.0165015.s002.png]
