# Supplementary material for: Filtration and Normalization of Sequencing Read Data in Whole-Metagenome Shotgun Samples
Source: PLoS One. 2016 Oct 19;11(10):e0165015. doi: 10.1371/journal.pone.0165015 (PMC5070866; doi:10.1371/journal.pone.0165015)
Supplement: S2 File — (RTF) [file pone.0165015.s004.rtf]

Call:
lm(formula = Norm_cov ~ poly(Genome_gc, 2, raw = TRUE), data = dfGC)

Residuals:
     Min       1Q   Median       3Q      Max 
-1.90188 -0.36113 -0.00795  0.47791  1.31293 

Coefficients:
                                  Estimate Std. Error t value Pr(>|t|)    
(Intercept)                      4.4739349  0.6721072   6.657 1.13e-10 ***
poly(Genome_gc, 2, raw = TRUE)1 -0.0808543  0.0274771  -2.943  0.00348 ** 
poly(Genome_gc, 2, raw = TRUE)2  0.0002736  0.0002670   1.024  0.30635    
---
Signif. codes:  0 ‘***’ 0.001 ‘**’ 0.01 ‘*’ 0.05 ‘.’ 0.1 ‘ ’ 1

Residual standard error: 0.6515 on 339 degrees of freedom
Multiple R-squared:  0.5217,	Adjusted R-squared:  0.5188 
F-statistic: 184.9 on 2 and 339 DF,  p-value: < 2.2e-16


Call:
lm(formula = Norm_cov ~ I(log(Genome_gc)), data = dfGC)

Residuals:
     Min       1Q   Median       3Q      Max 
-1.94827 -0.36833 -0.01565  0.49412  1.34047 

Coefficients:
                  Estimate Std. Error t value Pr(>|t|)    
(Intercept)        11.2116     0.5330   21.04   <2e-16 ***
I(log(Genome_gc))  -2.5925     0.1351  -19.18   <2e-16 ***
---
Signif. codes:  0 ‘***’ 0.001 ‘**’ 0.01 ‘*’ 0.05 ‘.’ 0.1 ‘ ’ 1

Residual standard error: 0.6519 on 340 degrees of freedom
Multiple R-squared:  0.5198,	Adjusted R-squared:  0.5183 
F-statistic:   368 on 1 and 340 DF,  p-value: < 2.2e-16


R-squared is almost the same as for the second degree polynomial model, but one predictor less. Both terms are highly significant (***).


Call:
lm(formula = Norm_cov ~ poly(GC, 2, raw = TRUE) + I(log(Genome_gc)), 
    data = dfGC)

Residuals:
     Min       1Q   Median       3Q      Max 
-0.98160 -0.29835 -0.04193  0.27703  1.05385 

Coefficients:
                           Estimate Std. Error t value Pr(>|t|)    
(Intercept)               8.564e+00  4.360e-01   19.64   <2e-16 ***
poly(GC, 2, raw = TRUE)1  1.201e-01  6.043e-03   19.88   <2e-16 ***
poly(GC, 2, raw = TRUE)2 -1.221e-03  6.117e-05  -19.97   <2e-16 ***
I(log(Genome_gc))        -2.550e+00  1.142e-01  -22.32   <2e-16 ***
---
Signif. codes:  0 ‘***’ 0.001 ‘**’ 0.01 ‘*’ 0.05 ‘.’ 0.1 ‘ ’ 1

Residual standard error: 0.4414 on 338 degrees of freedom
Multiple R-squared:  0.7811,	Adjusted R-squared:  0.7791 
F-statistic:   402 on 3 and 338 DF,  p-value: < 2.2e-16


Call:
lm(formula = Norm_cov ~ poly(GC, 3, raw = TRUE) + I(log(Genome_gc)), 
    data = dfGC)

Residuals:
     Min       1Q   Median       3Q      Max 
-0.75402 -0.24510 -0.00003  0.17802  1.23096 

Coefficients:
                           Estimate Std. Error t value Pr(>|t|)    
(Intercept)               6.256e+00  4.181e-01   14.97   <2e-16 ***
poly(GC, 3, raw = TRUE)1  3.159e-01  1.753e-02   18.02   <2e-16 ***
poly(GC, 3, raw = TRUE)2 -5.729e-03  3.894e-04  -14.71   <2e-16 ***
poly(GC, 3, raw = TRUE)3  3.080e-05  2.637e-06   11.68   <2e-16 ***
I(log(Genome_gc))        -2.568e+00  9.654e-02  -26.60   <2e-16 ***
---
Signif. codes:  0 ‘***’ 0.001 ‘**’ 0.01 ‘*’ 0.05 ‘.’ 0.1 ‘ ’ 1

Residual standard error: 0.373 on 337 degrees of freedom
Multiple R-squared:  0.8442,	Adjusted R-squared:  0.8423 
F-statistic: 456.3 on 4 and 337 DF,  p-value: < 2.2e-16


Call:
lm(formula = Norm_cov ~ poly(GC, 4, raw = TRUE) + I(log(Genome_gc)), 
    data = dfGC)

Residuals:
     Min       1Q   Median       3Q      Max 
-0.62768 -0.23063 -0.07693  0.19946  1.24722 

Coefficients:
                           Estimate Std. Error t value Pr(>|t|)    
(Intercept)               8.661e+00  4.953e-01  17.485  < 2e-16 ***
poly(GC, 4, raw = TRUE)1 -4.244e-03  4.443e-02  -0.096    0.924    
poly(GC, 4, raw = TRUE)2  6.384e-03  1.606e-03   3.975 8.63e-05 ***
poly(GC, 4, raw = TRUE)3 -1.512e-04  2.365e-05  -6.395 5.39e-10 ***
poly(GC, 4, raw = TRUE)4  9.400e-07  1.215e-07   7.738 1.19e-13 ***
I(log(Genome_gc))        -2.482e+00  8.976e-02 -27.646  < 2e-16 ***
---
Signif. codes:  0 ‘***’ 0.001 ‘**’ 0.01 ‘*’ 0.05 ‘.’ 0.1 ‘ ’ 1

Residual standard error: 0.3441 on 336 degrees of freedom
Multiple R-squared:  0.8677,	Adjusted R-squared:  0.8658 
F-statistic: 440.8 on 5 and 336 DF,  p-value: < 2.2e-16


Optimal non-linear regression:
Formula: Norm_cov ~ f(GC, c(m, s, a, b)) + I(p1 * GC) + I(p2 * GC^2) + 
    I(p3 * GC^3) + I(d * log(Genome_gc))

Parameters:
     Estimate Std. Error t value Pr(>|t|)    
m   4.934e+01  8.295e-01  59.488  < 2e-16 ***
s   8.887e+00  1.222e+00   7.274 2.50e-12 ***
a   8.121e-01  1.132e-01   7.177 4.66e-12 ***
b   6.830e+00  3.975e-01  17.182  < 2e-16 ***
p1  2.643e-01  2.171e-02  12.173  < 2e-16 ***
p2 -5.291e-03  4.602e-04 -11.498  < 2e-16 ***
p3  3.188e-05  3.134e-06  10.173  < 2e-16 ***
d  -2.502e+00  8.580e-02 -29.164  < 2e-16 ***
---
Signif. codes:  0 ‘***’ 0.001 ‘**’ 0.01 ‘*’ 0.05 ‘.’ 0.1 ‘ ’ 1

Residual standard error: 0.3283 on 334 degrees of freedom

Number of iterations to convergence: 12 
Achieved convergence tolerance: 9.335e-06
