# Supplementary material for: Filtration and Normalization of Sequencing Read Data in Whole-Metagenome Shotgun Samples
Source: PLoS One. 2016 Oct 19;11(10):e0165015. doi: 10.1371/journal.pone.0165015 (PMC5070866; doi:10.1371/journal.pone.0165015)
Supplement: S1 Table — (DOC) [file pone.0165015.s006.doc]

S1 Table. Comparison of commonly used whole metagenome microbiome profiling methods with the proposed method.

| Software (or Method) name | Method | Publication | Abundance estimate of top microbial species (or clades) in a sample | GC normalization of coverage | Feature-length normalization | Filtration of false hits due to reads mapping to genomic islands |
| --- | --- | --- | --- | --- | --- | --- |
| - | TBLASTX GenBank search followed by contig assembly to create OTUs for community diversity assessment | Breitbart, 2002  [6] | No (only percent of the most abundant virus and the total number of viruses in the sample were estimated) | No | No  (Effects of hypothetical variation of average viral genome length on abundance estimates were considered ) | N/A (the study concentrated on viruses where this was not applicable) |
| MEGAN | Every read is assigned to a taxon based on top BLAST hits according to the lowest  common ancestor of these hits. | Huson, 2007 [10] | Yes | No | No | No |
| CARMA | Each read is classified into a higher order taxonomy based on searching for conserved Pfam domains and protein families. The identified gene fragments are classified into a taxonomy based on reconstruction of a phylogenetic tree of each matching  Pfam family. | Krause, 2008 [12] | Yes | No | No | No |
| MG-RAST | Raw reads or assemblies are given as input. Quality control filtering for nucleotide sequence data (removal of artificial duplicate reads, quality-based read trimming, length-based read trimming, screening for DNA of model organisms). Protein similarity search between predicted proteins and database proteins (for shotgun) and a nucleic-acid similarity search (for reads similar to 16S and 18S sequences) | Meyer, 2008 [14] | Yes | No | No | No |
| PhymmBL | Classification of reads based on interpolated Markov models to characterize variable length oligonucleotides representative of a phylogenetic group and supplemented by a BLAST search approach. | Brady, 2009 [8] | Yes | No | No | No |
| MetaPhyler | Phylogenetic marker genes are used as a taxonomic reference. Reads are assigned to each marker and the depth of coverage is calculated as the median for each taxonomic unit. Relative abundances are calculated based on the depth of coverage values. | Liu, 2011  [13] | Yes | No | Length of each  HSP (high-scoring segment pairs in BLAST), the  reference gene, and the taxonomic level are used to adjust the estimates. | No |
| MetaPhlAn | Assigning reads to clade-specific marker genes using BLASTn. Clades range in specificity from phyla to species. | Segata, 2012 [16] | Yes | No | Yes (using the lengths of clade markers) | No |
| PhyloPythiaS | Works in generic and sample-specific modes. Relies on read assignment to clades inferred from reference sequences. | Patil, 2012 [15] | Yes | No | No | No |
| HUMAnN | Uses individual metagenomic reads as inputs and produces abundance of each orthologous gene family, presence/absence of each pathway, and abundance of each pathway in the community. | Abubucker, 2012  [7] | Calculates gene/pathway abundances | No | Normalization for gene length | No |
| Genometa | Each read is aligned using a short read aligner ( e.g., Bowtie [Ref]) to the reference database of complete bacterial genomes. | Davenport, 2012  [9] | Yes | No | No | Clustering of mapped reads can be checked |
| Taxy | Relies of protein-based mixture models instead of commonly used oligonucleotides models. Reconstructing the overall Pfam domain frequencies in a metagenomic sample by a linear combination of genomic reference signatures. | Klingenberg, 2013  [11] | Yes | No | No | No |
| ShotMAP | Uses reads or assembled sequences and a protein family database for metagenomic annotation. | Sharpton, unpublished  (https://github.com/sharpton/shotmap) | Yes | No | Yes | No |
| Our proposed method | Assigning a weight to each read based on its GC content and GC content of the genome to which it maps, reporting GC-weighted read counts per Mb of reference, filtration of false bacterial hits occurring due to read mapping to genomic islands. | This publication | Yes | Yes | Yes | Yes |
