# Supplementary material for: Filtration and Normalization of Sequencing Read Data in Whole-Metagenome Shotgun Samples
Source: PLoS One. 2016 Oct 19;11(10):e0165015. doi: 10.1371/journal.pone.0165015 (PMC5070866; doi:10.1371/journal.pone.0165015)
Supplement: S3 Table — (DOC) [file pone.0165015.s008.doc]

**S3** Table. Top 30 bacteria found in sputum of a cystic fibrosis patient

| **Species** | **Raw reads** | **GC-normalized reads** | **GC-normalized reads per Mb of reference** |
| --- | --- | --- | --- |
| Pseudomonas aeruginosa | 348476 | 1458519 | 224455 |
| Delftia acidovorans | 4204 | 7367 | 1089 |
| Thioalkalivibrio sulfidophilus | 1137 | 2836 | 819 |
| Rothia mucilaginosa | 1148 | 1799 | 794 |
| Pseudomonas stutzeri | 1247 | 2842 | 624 |
| Streptococcus salivarius | 2174 | 1005 | 453 |
| Pseudomonas putida | 1347 | 2586 | 431 |
| Bordetella petrii | 538 | 1904 | 360 |
| Prevotella melaninogenica | 1913 | 909 | 287 |
| Rothia dentocariosa | 880 | 687 | 274 |
| Pseudomonas mendocina | 479 | 1242 | 236 |
| Veillonella parvula | 1063 | 484 | 227 |
| Staphylococcus epidermidis | 1237 | 524 | 205 |
| Alicycliphilus denitrificans | 131 | 878 | 182 |
| Streptococcus parasanguinis | 735 | 349 | 162 |
| Acidovorax sp. | 218 | 719 | 162 |
| Leptotrichia buccalis | 883 | 347 | 141 |
| Azotobacter vinelandii | 196 | 688 | 128 |
| Pseudomonas fluorescens | 285 | 658 | 97 |
| Streptococcus thermophilus | 365 | 165 | 91 |
| Burkholderia gladioli | 154 | 732 | 90 |
| Streptococcus oralis | 342 | 166 | 85 |
| Acidovorax ebreus | 53 | 290 | 76 |
| Streptococcus pneumoniae | 341 | 156 | 73 |
| Streptococcus mitis | 322 | 151 | 70 |
| Ralstonia pickettii | 105 | 329 | 64 |
| Lactobacillus rhamnosus | 350 | 190 | 64 |
| Achromobacter xylosoxidans | 159 | 409 | 58 |
| Klebsiella pneumoniae | 299 | 259 | 48 |
| Streptococcus pseudopneumoniae | 195 | 89 | 41 |
